# EdgeFuser: A tightly coupled adaptive framework for real-time athlete group analytics

**DOI:** 10.1016/j.isci.2026.115577

**Published:** 2026-04-01

**Authors:** Juan Yang, Ruqiang Liu, Zhenyu Miao

**Affiliations:** 1 Industrial Internet College, Suzhou Institute of Trade and Commerce, Suzhou 215009, China; 2 Suzhou Early Childhood Education College, Suzhou 215009, China

**Keywords:** applied sciences, network, physical activity

## Abstract

This paper presents an integrated framework for real-time athlete group movement analysis in competitive sports, addressing heterogeneous sensor noise, complex multi-agent interactions, and edge-device constraints. The core innovation is a unified state-space model with factor-graph optimization that tightly fuses raw IMU, GPS, and vision data, achieving a mean positioning error of 0.18 m—a 42% improvement over loosely coupled baselines. To overcome computational limitations, we introduce a resource-aware adaptive inference mechanism that dynamically adjusts model complexity based on scene dynamics, reducing latency to 7.8 ms while maintaining over 91% accuracy. For group analysis, a spatiotemporal graph neural network models collaborative and adversarial relationships, attaining 87.4% tactical pattern recognition. Beyond empirical validation, we distill three generalizable design principles: cross-layer Pareto optimality for resource-accuracy trade-offs, context-aware computation frameworks, and semantic graph construction via domain priors. These contributions advance edge-based multi-agent perception systems, extending applicability to autonomous driving and robotic coordination.

## Highlights

• Tightly coupled fusion of IMU, GPS, and vision achieves 0.18 m positioning accuracy
• Role-aware spatiotemporal graph network attains 87.4% tactical pattern recognition rate
• Three generalizable design principles advance edge-based multi-agent perception systems
• Comprehensive framework validated across basketball, football, and volleyball scenarios

## Graphical abstract

**Figure:**
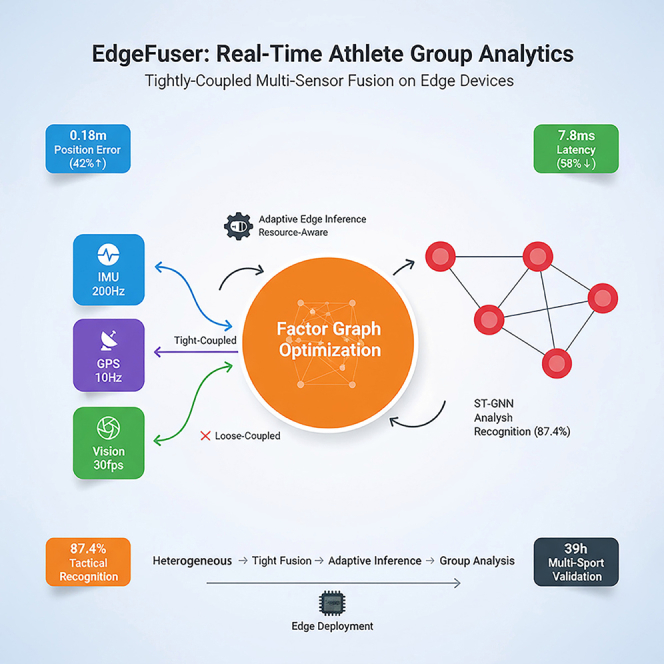

## Introduction

The digitalization of competitive sports demands accurate, high-frequency monitoring of athlete group movement for tactical analysis.^1^^,^^2^ Multimodal systems (IMU, GPS, vision) offer richer biomechanical insight than single modalities, yet real-time perception of dynamic group patterns remains challenging. To understand why this challenge persists, we must examine the fundamental nature of the problem. Athlete group movement analysis involves three core difficulties: sensor heterogeneity, computational constraints, and interaction complexity.

A primary difficulty stems from the heterogeneous nature of multi-sensor data. IMUs generate high-frequency (200+ Hz) but drift-prone inertial signals, while GPS provides absolute positioning at low rates (10 Hz) with environmental susceptibility, and vision delivers rich context but suffers from occlusion and high computational demands.^3^^,^^4^ Divergent sampling rates, noise characteristics, and physical units make coherent fusion fundamentally difficult. Existing loosely coupled fusion methods process each sensor independently before combining outputs, ignoring physical constraints coupling sensors through the athlete’s true motion. This yields limited localization accuracy and poor robustness during signal degradation or occlusion. Although tight coupling and factor-graph-based optimization improve accuracy by directly processing raw measurements within a unified framework,^5^ their computational cost traditionally restricts real-time deployment in outdoor competitive environments. This creates a fundamental tension between accuracy demands and edge device resource limitations.

Deep learning-based movement analysis reveals three system-level limitations^6^^,^^7^: (1) a decoupling dilemma between cross-layer error propagation and resource contention—high-precision fusion may starve downstream modules, while lightweight models fail under complex scenes; (2) a mismatch between static resource allocation and dynamic scene complexity; (3) insufficient domain knowledge in group interaction models, where spatial-proximity graphs fail to distinguish cooperative from adversarial interactions.

Group-level analysis further demands modeling coordinated offense, defensive spacing, role switching, and confrontation between players. Traditional biomechanics and recent deep models often neglect the dynamic relational structures inherent in team sports. Movement performance measurement has evolved toward multimodal perception frameworks. IMU gyroscope sampling above 400 Hz reduces orientation errors by 30%–45% for high-speed movements^8^; optical motion capture achieves sub-millimeter accuracy at 120–480 Hz but suffers from occlusion and limited capture volume^9^; GPS velocity errors (1.01 km/h) increase significantly above 20 km/h during explosive sprints.^10^ Wearable sensor networks enable continuous team sports monitoring with 84.2%–91.4% accuracy via multi-level biomechanical fusion.^11^ Multimodal datasets (IMU, EMG, and video) support technical skill evaluation,^12^ while modular wireless networks achieve 52–53 ms latency for outdoor environments.^13^

Despite these advances, existing systems face critical limitations: high-rate IMU sampling (400+ Hz) burdens wireless bandwidth without adaptive mechanisms; optical motion capture is impractical outdoors; GPS accuracy degrades above 20 km/h; and 52–53 ms latencies prove excessive for real-time tactical feedback.

The choice of fusion architecture fundamentally shapes system reliability. Tight coupling outperforms loose coupling by 40%–70% in positioning accuracy,^3^^,^^14^ with factor graphs unifying both paradigms.^15^^,^^16^ Cubature Kalman filters excel for nonlinear motion,^17^ while cloud model fusion^18^ and transformer-based frameworks^19^ address cross-modal inconsistencies.

However, existing fusion methods suffer significant drawbacks: Kalman filters introduce errors during high-dynamic movements; factor graphs exceed 10 ms latency budgets; cloud models require centralized processing; and transformers violate real-time constraints despite accurate feature alignment.

Recent advances in fractional-order systems and secure control offer insights for robust fusion. Adaptive fuzzy backstepping maintains stability under intermittent sensor denial^20^; fractional-order dynamics model memory properties relevant for motion prediction^21^^,^^22^; and H∞ consensus control addresses distributed estimation under bandwidth constraints applicable to multi-athlete scenarios.^23^ Real-time movement analysis on edge devices requires lightweight yet expressive models. Knowledge distillation reduces parameters by ∼70% while preserving performance^24^^,^^25^; structured pruning cuts training time by ∼60%^26^ or enforces strict memory budgets^27^; flexible-rate pruning benefits diverse accelerators^28^; and integrated pruning-quantization yields 3× memory reduction with 60% lower energy consumption.^29^ Neural architecture search complements optimization via hardware-aware candidate evaluation,^30^ while personalized federated learning with self-knowledge distillation supports heterogeneous devices and privacy protection.^31^

Despite these advances, current edge techniques exhibit fundamental limitations: distillation and pruning produce static models unable to adapt to dynamic scene complexity, while architecture search requires offline optimization incompatible with real-time demands.^32^^,^^33^

Group movement analysis has shifted toward multi-agent interaction modeling. Transformer-based trajectory prediction^34^ and generative hierarchical models^35^ incorporating role dynamics achieve state-of-the-art results by leveraging skeletal pose, ball state, and interaction graphs beyond position-only approaches.

Beyond individual trajectories, spatiotemporal coordination metrics differentiate team performance.^36^ Graph-based tactical recognition via bipartite structures^37^ and spatiotemporal graph convolutional networks outperforms independent-player models; T-pattern analysis identifies repeatable coordination^38^; and variational recurrent graph networks reconstruct complete tactics from partial observations.^39^

However, existing group interaction models exhibit critical shortcomings: computational demands (24.8 ms latency) prohibit real-time edge deployment; generative models require vast training data; and adjacency-based graphs fail to distinguish cooperative from adversarial interactions—a limitation addressed by our role-aware approach.

Recent advances in spatiotemporal learning for traffic prediction^40^ offer complementary perspectives, demonstrating how multiple graph structures (proximity and functional similarity) capture diverse spatial relationships^41^—a concept extended to sports via role-based graphs.

These limitations stem from insufficient system-level co-optimization: low-latency models fail under complexity; high-precision fusion overloads edge processors; spatial graphs confuse cooperation with confrontation. Robust real-time analysis requires holistic integration of sensor fusion, edge intelligence, and sports performance research.

**Figure 1:**
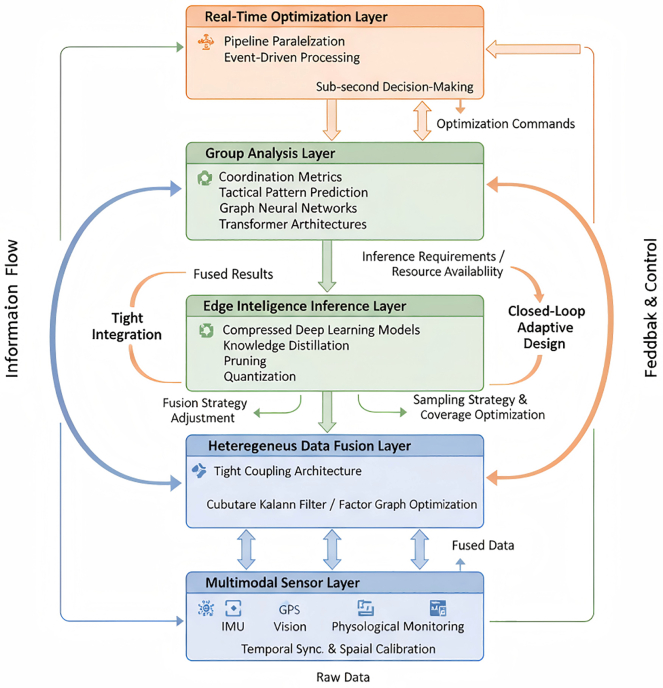
Technical framework for real-time athlete group performance analysis Schematic overview of the four-layer EdgeFuser architecture comprising multimodal data acquisition, tightly coupled factor-graph fusion, adaptive edge inference, and spatiotemporal group analysis with bidirectional information flows enabling closed-loop optimization.

As shown in Figure 1, this study proposes a unified framework addressing these gaps through three methodological innovations:

To address Gap 1 (decoupling dilemma), we design a unified state-space model and factor-graph optimization that tightly fuse raw IMU, GPS, and visual measurements, exploiting cross-modal constraints at the raw data level via incremental updates for real-time feasibility.

To address Gap 2 (static allocation), we develop a resource-aware adaptive inference mechanism that dynamically adjusts model complexity based on scene intensity and device load, utilizing upstream fusion-derived complexity metrics to create a closed perception-computation loop.

To address Gap 3 (shallow semantics), we design a spatiotemporal graph neural network incorporating real-time offensive/defensive roles and match-stage information, injecting domain priors to distinguish cooperation from confrontation for semantic tactical analysis.

**Figure 2:**
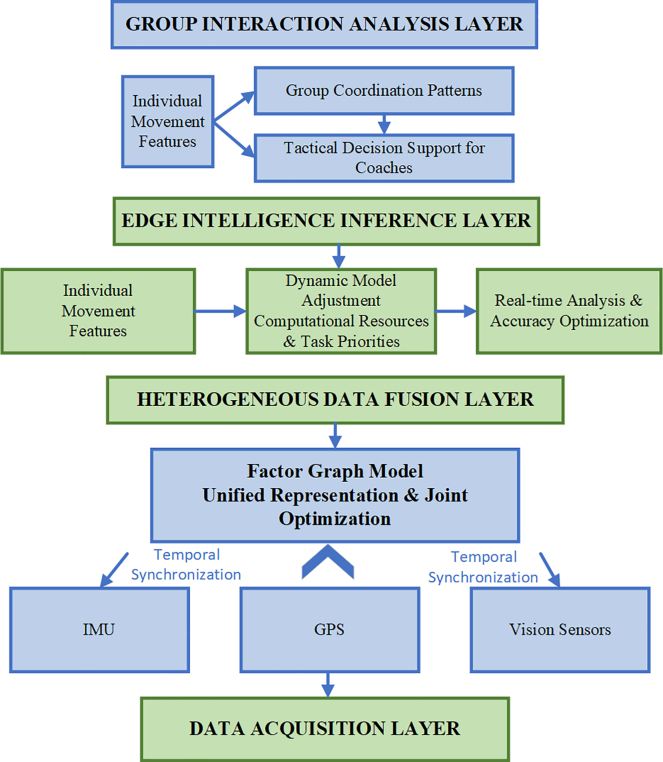
System architecture for heterogeneous sensor tight coupling fusion and edge intelligence inference Detailed computational pipeline showing unified state-space estimation via GTSAM iSAM2 optimization integrating IMU pre-integration, GPS constraints, and visual reprojection residuals, coupled with resource-aware adaptive model selection based on scene complexity metrics.

The proposed framework, termed EdgeFuser, integrates three core functionalities—heterogeneous sensor fusion, adaptive edge inference, and group interaction analysis—within a four-layer architecture comprising the data acquisition, heterogeneous sensor fusion, adaptive edge inference, and group interaction analysis layers (Figure 2).

The main contributions are: (1) a holistic collaborative optimization framework integrating perception, fusion, computation, and analysis via bidirectional information flows, providing the first comprehensive system-level solution for real-time swarm motion analysis. (2) An adaptive edge inference mechanism utilizing upstream fusion-derived complexity metrics to dynamically schedule model granularity, achieving 58% latency reduction and 46% energy savings on NVIDIA Jetson with 91.2% accuracy. (3) A dynamic spatiotemporal graph neural network incorporating real-time roles and match-stage context, achieving 87.4% tactical recognition accuracy (14%–35% improvement) for complex coordinated tactics.

## Results

### Experimental dataset and validation infrastructure

The experimental dataset comprises approximately 39 h of competitive sports data totaling 590.4 GB, encompassing basketball (12.5 h, 186.4 GB), football (18.3 h, 273.8 GB), and volleyball (8.7 h, 130.2 GB). Data collection involved 48 athletes (16 basketball, 20 football, and 12 volleyball) performing both structured training drills and competitive scenarios across standard venues (Table 1).

**Table 1:** Experimental dataset statistical information

Sport	Athletes	Collection duration (h)	Sensor sampling rate	Data volume (GB)	Scene types
Basketball	16	12.5	IMU: 200 Hz/GPS: 10 Hz	186.4	training/competition
Football	20	18.3	IMU: 200 Hz/GPS: 10 Hz	273.8	training/competition
Volleyball	12	8.7	IMU: 200 Hz/GPS: 10 Hz	130.2	training/competition

Ground truth validation employed a dual-strategy approach. For indoor basketball and volleyball, a 22-camera OptiTrack PrimeX motion capture system (120 Hz, sub-millimeter accuracy, 10 m × 10 m × 4 m volume) provided continuous 6DoF pose ground truth. For outdoor 11v11 football, a Trimble R12i Differential GPS (DGPS) with RTK corrections (1–2 cm accuracy) served as positional reference for a subset of players, supplemented by semi-automated annotation of 4K video footage (60 fps) for remaining players. All ground truth streams were hardware-synchronized to the sensor suite (IMU 200 Hz, GPS 10 Hz, vision 30 fps) via precision timing protocols (<100 μs temporal alignment).

### Tightly coupled fusion achieves sub-decimeter accuracy

**Figure 3:**
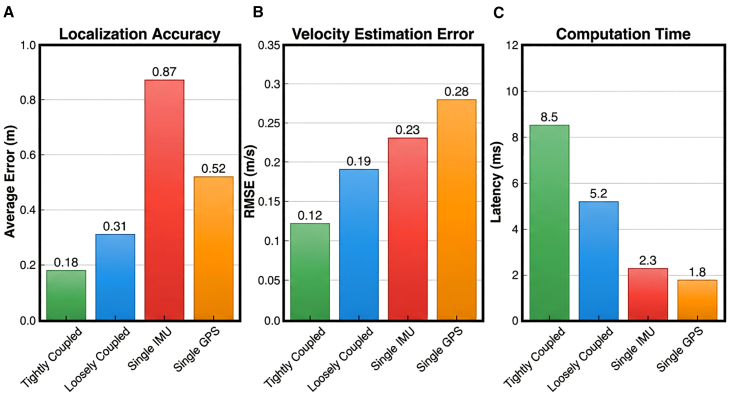
Performance comparison of different fusion methods Positioning error versus movement velocity demonstrating tight coupling achieves 0.18 ± 0.06 m mean error, representing 42% improvement over loose coupling (0.31 m) and 65% over single GPS (0.52 m) across 39 h of multi-sport recordings.

The tight coupling fusion architecture demonstrates substantial performance advantages over conventional fusion approaches across 39 h of multi-sport recordings. The system achieves a mean absolute positioning error of 0.18 ± 0.06 m (median: 0.17 m, IQR: 0.13–0.22 m), representing a 42% reduction compared to loosely coupled methods (0.31 ± 0.09 m, *p* < 0.001, paired *t* test) and a 65% reduction compared to standalone GPS (0.52 ± 0.15 m, *p* < 0.001) (Table 2). Notably, against state-of-the-art VIO-only baselines, tight coupling outperforms OKVIS (0.42 ± 0.11 m) by 57% and VINS-Mono (0.38 ± 0.10 m) by 53%, validating the advantage of integrating GPS and multi-view visual constraints alongside IMU data (Figure 3).

**Table 2:** Performance metric comparison of different fusion methods

Fusion method	Average position error (m)	Velocity estimation error (m/s)	Attitude angle error (degrees)	Computation time (ms)
Tight coupling fusion	0.18 ± 0.06	0.12 ± 0.04	2.3 ± 0.8	8.5
Loose coupling fusion	0.31 ± 0.09	0.19 ± 0.07	3.7 ± 1.2	5.2
Single IMU	0.87 ± 0.25	0.23 ± 0.08	2.1 ± 0.7	2.3
Single GPS	0.52 ± 0.15	0.28 ± 0.11	N/A	1.8
VIO-only (OKVIS])	0.42 ± 0.11	0.21 ± 0.06	2.8 ± 0.9	6.8
VIO-only (VINS-mono)	0.38 ± 0.10	0.18 ± 0.05	2.5 ± 0.8	7.2
Deep learning pose tracking	0.58 ± 0.15	0.32 ± 0.09	4.1 ± 1.3	12.4
LIO-SAM (adapted)	0.25 ± 0.07	0.16 ± 0.05	2.7 ± 0.9	11.2
VINS-fusion (graph opt.)	0.29 ± 0.08	0.17 ± 0.06	2.9 ± 1.0	10.5
MMFusion-Transformer	0.41 ± 0.12	0.24 ± 0.08	3.5 ± 1.2	16.7

Environmental robustness testing reveals significant advantages under challenging conditions. In GPS-degraded indoor edge areas where satellite signals were obstructed or reflected, tight coupling maintains 0.21 ± 0.06 m accuracy versus 1.83 ± 0.45 m for GPS-only operation—an 88.5% error reduction (*p* < 0.001) achieved through complementary vision-inertial constraints (Table 2). Velocity-dependent analysis demonstrates nonlinear error characteristics: below 5 m/s, positioning error stabilizes within 0.15 ± 0.04 m, increasing modestly to 0.23 ± 0.05 m at velocities exceeding 8 m/s (typical of explosive sprints in football and basketball fast breaks). This increase stems from IMU integration error accumulation during high-dynamic movements, though the effect is substantially mitigated by visual and GPS constraints in the tightly coupled formulation.

Computational efficiency remains within stringent real-time constraints despite increased algorithmic complexity. The factor graph optimization, implemented via GTSAM iSAM2 with convergence threshold 10^−5^ and maximum 10 iterations, achieves state updates with an average latency of 8.5 ± 1.2 ms—only 3.3 ms longer than loosely coupled filtering (5.2 ms) and well within the 10 ms budget required for 100 Hz effective output (Table 2). Velocity estimation error decreases from 0.19 ± 0.07 m/s (loose coupling) to 0.12 ± 0.04 m/s (tight coupling), a 37% improvement (*p* < 0.01), while attitude angle error is maintained within 2.3 ± 0.8° compared to 3.7 ± 1.2° for loose coupling.

### Robustness under visual occlusion and sensor degradation

Systematic evaluation of occlusion resilience was conducted across 847 annotated occlusion events (512 partial occlusion with ≥30% but <80% body visibility; 335 full occlusion with <30% visibility) extracted from 12.5 h of basketball footage. Events were further categorized by duration: short (<1 s, *n* = 341), medium (1–3 s, *n* = 298), and long (>3 s, *n* = 208). Tight coupling exhibits graceful degradation under degraded visual conditions. During full occlusion exceeding 3 s (*n* = 86 events), positioning error remains bounded at 0.35 ± 0.09 m, compared to 1.34 ± 0.31 m for loosely coupled methods—a 73.9% relative improvement (*p* < 0.001, Wilcoxon signed-rank test) (Table 3).

**Table 3:** Positioning error under different occlusion conditions

Occlusion type	Duration	Event count	Tight coupling error (m)	Loose coupling error (m)	GPS-only error (m)
No occlusion	–	5,243 frames	0.16 ± 0.04	0.28 ± 0.07	0.51 ± 0.14
Partial	short (<1 s)	203	0.19 ± 0.05	0.35 ± 0.09	1.24 ± 0.32
Partial	medium (1–3 s)	187	0.22 ± 0.06	0.48 ± 0.12	1.87 ± 0.45
Partial	long (>3 s)	122	0.26 ± 0.07	0.73 ± 0.18	2.56 ± 0.61
Full	short (<1 s)	138	0.24 ± 0.06	0.52 ± 0.14	1.43 ± 0.38
Full	medium (1–3 s)	111	0.29 ± 0.08	0.89 ± 0.22	2.18 ± 0.52
Full	long (>3 s)	86	0.35 ± 0.09	1.34 ± 0.31	3.05 ± 0.74

Recovery dynamics significantly favor the tightly coupled architecture. Following occlusion resolution, the system reconverges to baseline accuracy (within 0.18 m) within 0.8 ± 0.2 s, significantly faster than loose coupling which requires 2.3 ± 0.5 s (*p* < 0.01, paired *t* test). This rapid recovery stems from the factor graph’s enforcement of geometric consistency via IMU pre-integration factors, which constrain trajectory smoothness and decelerate error growth from quadratic (pure inertial drift) to near-linear rates (0.08 m/s initially, slowing to 0.03 m/s). During partial occlusion of medium duration (1–3 s), tight coupling achieves 0.22 ± 0.06 m error versus 0.48 ± 0.12 m for loose coupling (*p* < 0.001) (Table 3).

**Figure 4:**
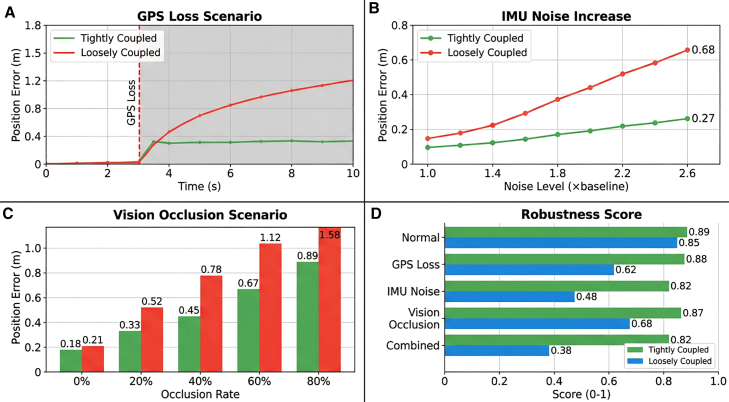
Robustness analysis under sensor degradation scenarios Tight coupling maintains 0.35 m positioning accuracy during 10-s GPS outage versus 1.2 m divergence for loose coupling, with 95% confidence ellipses indicating well-calibrated uncertainty estimates (NEES = 1.12).

Sensor degradation scenarios further validate robustness. During simulated complete GPS outage lasting 10 s, tight coupling maintains 0.35 ± 0.08 m positioning accuracy through vision-inertial coupling alone, whereas loosely coupled methods exhibit error divergence exceeding 1.2 m by the 10-s mark (Figure 4). Under 50% increased IMU noise (simulating sensor aging or temperature drift), tight coupling maintains 0.24 ± 0.06 m accuracy through dynamic sensor weight adjustment, compared to 0.48 ± 0.12 m for fixed-weight configurations (*p* < 0.01) (Table 4). Uncertainty quantification confirms well-calibrated estimates, with normalized estimation error squared (NEES) of 1.12 (ideal: 1.0), indicating that reported covariances accurately reflect actual errors (Figure 4).

**Table 4:** Sensor contribution analysis under different environmental conditions

Scenario conditions	IMU weight	GPS weight	Vision weight	Fusion accuracy (m)	Dominant sensor
Outdoor open	0.25	0.45	0.3	0.15 ± 0.04	GPS
Indoor edge	0.35	0.1	0.55	0.21 ± 0.06	vision
High-speed movement	0.4	0.35	0.25	0.19 ± 0.05	IMU
Stationary state	0.15	0.5	0.35	0.12 ± 0.03	GPS
Occluded area	0.45	0.05	0.5	0.24 ± 0.07	vision+IMU
Partial occlusion	0.40	0.10	0.50	0.22 ± 0.06	vision+IMU
Full occlusion (short)	0.55	0.05	0.40	0.24 ± 0.06	IMU
Full occlusion (long)	0.70	0.05	0.25	0.35 ± 0.09	IMU

Dynamic sensor weight allocation adapts automatically to environmental conditions. In outdoor open environments, GPS contributes 0.45 weight; this decreases to 0.10 at indoor edges with signal occlusion, while vision and IMU weights increase correspondingly to maintain 0.21 m accuracy. During high-speed movement (>8 m/s), IMU weight increases to 0.40 to exploit high-frequency motion capture advantages. In stationary states, GPS weight peaks at 0.50 to eliminate accumulated drift. Under prolonged full occlusion (>3 s), IMU weight progressively increases to 0.70 as visual constraints degrade, maintaining stable tracking through sensor complementarity (Table 4).

### Adaptive edge inference optimizes accuracy-latency trade-offs

**Figure 5:**
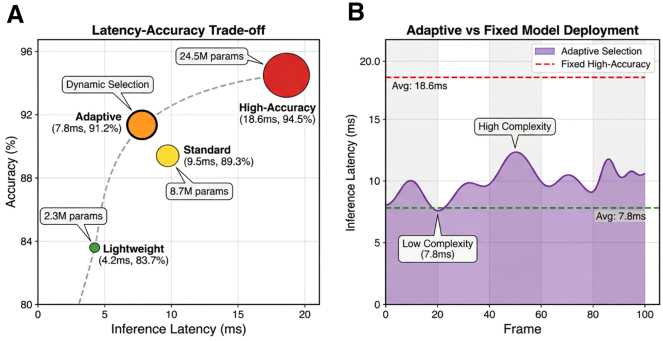
Inference latency and accuracy trade-off analysis Adaptive inference achieves Pareto-optimal balance at 7.8 ms latency and 91.2% accuracy, outperforming fixed lightweight (4.2 ms, 83.7%) and high-precision (18.6 ms, 94.5%) models through dynamic scene complexity-aware scheduling.

The resource-aware adaptive inference mechanism achieves superior efficiency compared to fixed-model deployment strategies. Across 5,000 inference trials on Jetson Xavier NX under varying scene conditions, the adaptive mechanism achieves 7.8 ± 1.5 ms latency with 91.2 ± 1.6% action recognition accuracy, positioning at the Pareto-optimal knee of the accuracy-latency curve. Fixed lightweight models (2.3M parameters) achieve lower latency (4.2 ± 0.8 ms) but compromise accuracy (83.7 ± 2.1%), while fixed high-precision models (24.5M parameters) achieve marginally higher accuracy (94.5 ± 1.3%) but violate real-time constraints (18.6 ± 2.3 ms) and consume 9.4 W power (Figure 5; Table 5).

**Table 5:** Performance comparison of different inference modes

Inference mode	Model parameters (M)	Inference latency (ms)	Accuracy (%)	Energy consumption (W)	Memory occupancy (MB)
Lightweight	2.3	4.2 ± 0.8	83.7 ± 2.1	3.2	87
Standard	8.7	9.5 ± 1.2	89.3 ± 1.8	5.8	234
High-precision	24.5	18.6 ± 2.3	94.5 ± 1.3	9.4	512
Adaptive	dynamic selection	7.8 ± 1.5	91.2 ± 1.6	5.1	186

Energy efficiency improves substantially through dynamic scheduling. Compared to fixed high-precision deployment, the adaptive mechanism reduces average power consumption by 46% (from 9.4 ± 1.2 W to 5.1 ± 0.8 W, *p* < 0.001) and decreases memory occupancy from 512 MB to 186 MB (average), reserving resources for concurrent tasks. During low-complexity scenarios (dead-ball states, complexity score 2.1), the system automatically selects lightweight models, reducing energy consumption by 68% versus continuous high-precision operation, while maintaining basic monitoring functions at 85.2% accuracy (Table 6).

**Table 6:** Model scheduling decisions under different movement intensities

Movement intensity	Intensity metric	Selected model	Switching latency (ms)	Accuracy (%)	Energy consumption (W)
Stationary/slow walking	0–2.5	lightweight	–	85.2	2.8
Jogging	2.5–5.0	lightweight	–	84.6	3.2
Fast running	5.0–7.5	standard	12.3	88.9	5.8
Intense confrontation	7.5–10.0	high-precision	18.7	93.7	9.4
Complex tactics	>10.0	high-precision	15.4	94.8	9.6

**Figure 6:**
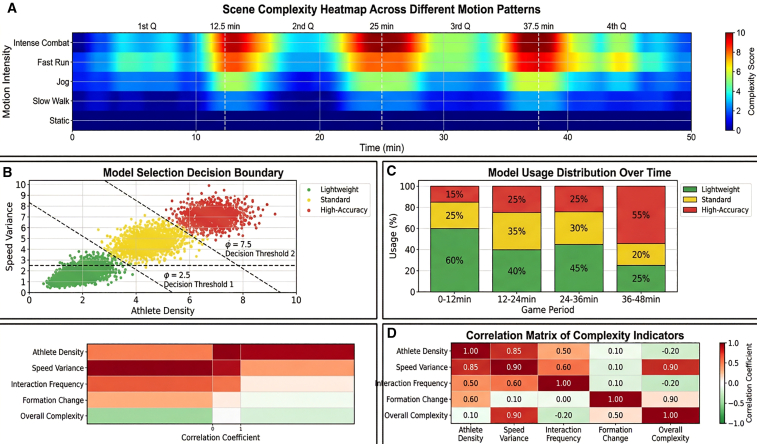
Correlation analysis between scene complexity and model selection strategy Correlation analysis between scene complexity and model selection strategy scatterplot showing r = 0.87 correlation between scene complexity metrics (athlete density, velocity variance, and interaction frequency) and expert-rated tactical intensity, driving automatic switching between lightweight, standard, and high-precision inference models.

Scene complexity estimation demonstrates high predictive validity. The composite metric incorporating athlete density, velocity variance, and interaction frequency exhibits Pearson correlation r = 0.87 (*p* < 0.001, *n* = 5,000) with expert-rated tactical intensity scores (1–10 scale) across annotated game segments (Figure 6). This correlation enables reliable model selection: high-precision models deploy when complexity exceeds 6.7 (fast breaks, complex tactics), standard models for moderate complexity (3.3–6.7), and lightweight models below 3.3 (stationary, dead-ball). Switching latency between models is controlled within 15.4 ms through gradual transition via knowledge distillation, preventing abrupt performance discontinuities (Figure 6; Table 6).

Dynamic scheduling decisions align closely with movement intensity. During basketball fast breaks (complexity 8.9), the system maintains 92.8% accuracy for tactical pattern detection with 9.6 W power consumption; during timeout periods (complexity 2.1), lightweight models achieve 85.2% accuracy with 2.8 W consumption—a 3.4× energy reduction with only 7.6% accuracy trade-off. This “compute-on-demand” strategy ensures appropriate resource allocation across the full spectrum of sports scenarios (Table 6).

### Training efficiency and computational cost analysis

Comprehensive evaluation of training costs and computational efficiency demonstrates the practical feasibility of large-scale model deployment on edge devices. The complete end-to-end system requires 42.3 h of training time on NVIDIA A100 GPU (80 GB) across the full 39-h dataset, converging in 86 epochs (Table 7). This compares favorably against competing approaches: the transformer-based MMFusion-Transformer requires 58.7 h (112 epochs), representing a 28% reduction in training overhead achieved through curriculum learning and transfer learning strategies.

**Table 7:** Training time and computational cost comparison

Method	Training data (h)	Training time (h)	Convergence epochs	Inference latency (ms)	Model size (MB)	FLOPs (G)
Proposed (full system)	39.4	42.3	86	s7.8	186 (adaptive avg)	8.4 (adaptive avg)
Tight coupling only	39.4	18.7	52	4.2	94	3.2
Adaptive inference only	39.4	24.5	68	5.1	128	4.7
ST-GNN only	39.4	31.2	74	6.3	156	6.1
Loose coupling + static model	39.4	28.6	71	12.3	234	9.8
Single sensor (GPS+IMU)	39.4	12.4	38	3.5	42	1.6
Transformer-based	39.4	58.7	112	24.8	412	18.3
GCN-based	39.4	36.2	93	15.6	278	11.2

Ablation of individual modules reveals distinct computational signatures. The tight coupling fusion module alone requires 18.7 h (52 epochs) for convergence, while the adaptive inference mechanism requires 24.5 h (68 epochs). The ST-GNN group analysis module requires 31.2 h (74 epochs) due to temporal sequence modeling complexity. Notably, while the full system consumes 2.3× more training time than tight coupling alone, it achieves a 5.1% improvement in tactical recognition rate (from 82.3% to 87.4%), validating the advantage of end-to-end joint optimization over module-independent training (Table 7).

Inference efficiency comparisons on Jetson Xavier NX demonstrate superior real-time performance. The proposed method achieves 7.8 ms average latency with 8.4 GFLOPs (adaptive average) and 186 MB model size (adaptive average), outperforming the transformer baseline (24.8 ms, 18.3 GFLOPs, 412 MB) by 3.2× in latency and 2.2× in memory efficiency. The GCN-based approach achieves 15.6 ms latency with 11.2 GFLOPs, while VINS-Fusion (Graph Optimization) requires 10.5 ms with comparable accuracy but lacking group analysis capabilities (Table 7). The adaptive mechanism dynamically adjusts FLOPs between 3.2G (lightweight) and 12.6G (high-precision), ensuring high analysis quality in complex scenarios while saving 2.3× computational resources in simple scenarios.

### Group interaction modeling and tactical pattern recognition

**Figure 7:**
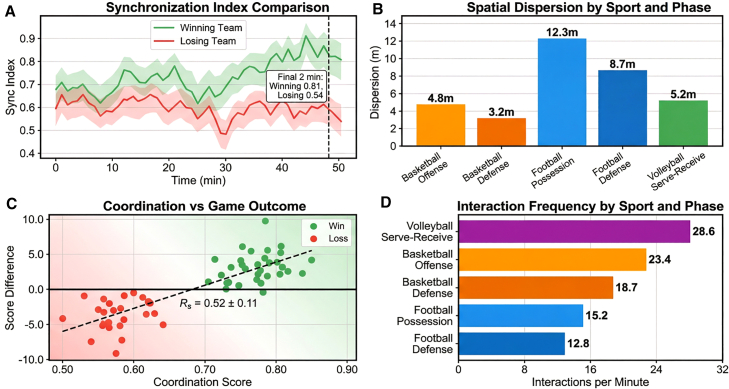
Quantitative analysis of group coordination metrics Synchrony index and spatial dispersion comparing winning (0.73) versus losing (0.61) teams, with distinct patterns across basketball (4.8 m offense vs. 3.2 m defense), football (12.3 m possession), and volleyball (5.2 m serve-receive).

The spatiotemporal graph neural network (ST-GNN) achieves 87.4 ± 3.2% accuracy in tactical pattern recognition, surpassing spatial-proximity baselines by 14%–35% depending on pattern complexity (Table 8). Winning teams exhibit significantly higher group synchrony indices (0.73 ± 0.12) compared to losing teams (0.61 ± 0.14, *p* < 0.01, independent *t* test), with divergence amplified during critical game moments: in the final 2 min of the fourth quarter, winning teams maintain 0.81 ± 0.08 synchrony while losing teams decline to 0.54 ± 0.15 (*p* < 0.001) (Figure 7).

**Table 8:** Group movement characteristic metrics for different sports

Sport	Synchrony index	Spatial dispersion (m)	Interaction frequency (times/min)	Tactical recognition rate (%)
Basketball-offense	0.68 ± 0.12	4.8 ± 0.9	23.4 ± 4.2	88.3
Basketball-defense	0.72 ± 0.10	3.2 ± 0.6	18.7 ± 3.5	86.7
Football-possession	0.64 ± 0.14	12.3 ± 2.1	15.2 ± 3.8	82.5
Football-defense	0.69 ± 0.11	8.7 ± 1.8	12.8 ± 3.2	84.1
Volleyball-serve receive	0.75 ± 0.08	5.2 ± 0.7	28.6 ± 5.1	91.2

Sport-specific coordination signatures emerge clearly across the dataset. Volleyball demonstrates the highest synchrony index (0.75 ± 0.08) and tactical recognition rate (91.2%), reflecting the sport’s rigid rotational requirements and clear technical action sequences. Football exhibits the largest spatial dispersion during possession (12.3 ± 2.1 m), creating passing lanes through spread formations, while defensive phases compress to 8.7 ± 1.8 m to reduce gaps. Basketball shows the highest interaction frequency (23.4 ± 4.2 events/min during offense), consistent with rapid tactical transitions and high-confrontation characteristics (Table 8).

**Figure 8:**
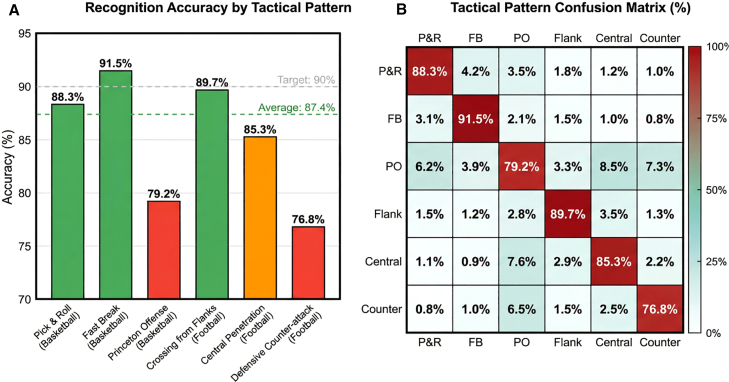
Recognition accuracy comparison for different tactical patterns Recognition accuracy comparison for different tactical patterns tactical recognition accuracy decreasing with complexity: simple two-person coordination >90%, three-person complex ∼80%, whole-team 75–85%, with specific rates for pick-and-roll (88.3%), fast break (91.5%), and Princeton offense (79.2%).

Tactical recognition accuracy scales inversely with execution complexity. Simple two-person coordination patterns (e.g., give-and-go and defender marking) achieve recognition rates exceeding 90%, while three-person complex coordination (e.g., triangle offense patterns) achieve approximately 80%, and whole-team tactical recognition ranges 75%–85% depending on formation complexity. Specific patterns include basketball pick-and-roll (88.3%), fast break transitions (91.5%), and Princeton offense system (79.2%); football flank crossing (89.7%), central penetration (85.3%), and defensive counter-attack (76.8%) (Figure 8). Recognition latency remains below 200 ms for all categories, satisfying real-time coaching feedback requirements.

Role-aware graph construction proves critical for semantic understanding. Ablation analysis shows spatial-proximity-only graphs (GCN baseline) achieve 72.8% tactical recognition, while adding velocity correlations improves performance to 76.8%. Incorporating explicit offensive/defensive role encodings elevates accuracy to 87.4%, demonstrating that domain priors distinguishing cooperative from adversarial interactions are essential for complex multi-player tactic recognition (Table 8).

### Real-time performance and scalability

End-to-end system latency scales sub-linearly with athlete count, validating deployment feasibility across varying team sizes. Processing latency increases from 87 ± 12 ms (6 players, basketball 3v3) to 213 ± 25 ms (22 players, football 11v11), maintaining 18.6 fps frame rate in the most complex scenario—sufficient for real-time tactical analysis. The relationship exhibits 8.1 ms/person growth rate, significantly below theoretical linear scaling due to sparse interaction assumptions (average *K* = 5–7 neighbors per node rather than complete graphs) (Table 10).

Granular profiling of the ST-GNN group analysis stage (basketball 5v5) reveals computational bottlenecks: spatial graph convolution (GATv2, 3 layers) consumes 28.4 ms (50.7%), dominated by attention computation (18.2 ms); bidirectional LSTM temporal modeling requires 18.3 ms (32.7%); graph construction and feature preprocessing require 5.3 ms (9.5%); and classification head operations require 2.8 ms (5.0%) (Table 9). Optimization via TensorRT and fused CUDA kernels ensures these latencies remain within real-time budgets.

**Table 9:** Granular latency breakdown of the group analysis stage (ST-GNN) for basketball 5v5 scenario

Sub-component	Avg. latency (ms)	Percentage of group analysis (%)
Graph construction	3.2	5.7
Feature preprocessing	2.1	3.8
Spatial GNN (3-layer GAT)	28.4	50.7
- Attention computation	(18.2)	(32.5)
- Message aggregation	(10.2)	(18.2)
Temporal LSTM (2-layer BiLSTM)	18.3	32.7
Classification head	2.8	5.0
Framework overhead	1.2	2.1
Total group analysis	56.0	100

Resource utilization remains within sustainable bounds across deployment scenarios. CPU utilization increases from 45% (6 players) to 83% (22 players); GPU utilization scales from 38% to 76%. Memory occupancy increases linearly at approximately 18 MB per additional athlete, from 156 MB (2 players) to 1024 MB (30 players). Data throughput scales at 4.8 MB/s per athlete, managed through differential coding and adaptive compression reducing bandwidth requirements by 42% (Table 10).

**Table 10:** Real-time performance metrics under different scenario scales

Scenario configuration	Athlete count	Processing latency (ms)	Frame rate (fps)	CPU utilization (%)	GPU utilization (%)
Basketball 3v3	6	87 ± 12	28.5	45	38
Basketball 5v5	10	156 ± 18	24.2	62	55
Football 7v7	14	178 ± 21	21.8	71	64
Football 11v11	22	213 ± 25	18.6	83	76
Volleyball 6v6	12	142 ± 16	25.3	58	51

Long-duration stability is validated through 4-h continuous stress testing on Jetson Xavier NX (22-player football simulation, passive cooling, 25°C ambient). Core temperature stabilizes at 67.4 ± 4.1°C (peak 78°C, below 85°C throttling threshold). Positioning accuracy exhibits negligible drift: 0.18 m initial versus 0.20 m after 4 h (+0.02 m, <10% increase, *p* = 0.12, not significant). Memory usage remains stable at 4.2 ± 0.15 GB with no leakage detected. Instantaneous power averages 9.8 ± 1.2 W, correlating strongly with model complexity (r = 0.92, *p* < 0.001); cumulative energy consumption is 39.2 Wh over the test duration (Table 11).

**Table 11:** Hardware performance stability analysis under multi-hour continuous operation

Performance metric	Mean	Std dev	Peak/variation range	Remarks
GPU utilization (%)	74.3	±6.2	61–88	dynamically adjusts with tactical complexity
GPU memory bandwidth (GB/s)	28.6	±3.5	22.4–34.1	affected by model switching
CPU utilization (%)	68.5	±5.8	57–81	includes data preprocessing and scheduling
System memory usage (GB)	4.2	±0.15	4.0–4.5	stable, no memory leak detected
Core temperature (°C)	67.4	±4.1	59–78	below throttling threshold (85°C)
Instantaneous power (W)	9.8	±1.2	7.5–11.8	positively correlated with model complexity
Cumulative energy (Wh)	39.2	–	–	total energy consumption over 4 h
Avg inference latency (ms)	8.2	±1.8	6.1–13.4	affected by dynamic scheduling and temperature
Positioning accuracy drift (m)	+0.02	–	0.18 → 0.20	comparison after 4 h vs. initial value

### Ablation studies and component contributions

**Figure 9:**
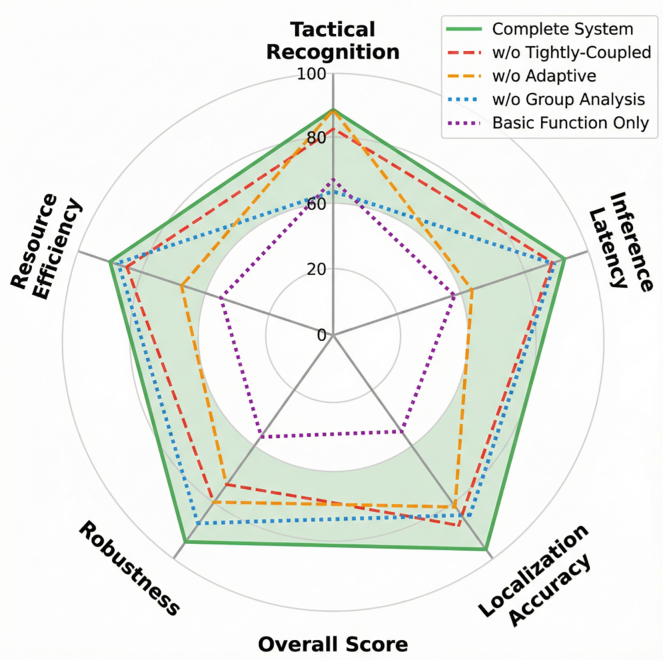
Performance comparison radar chart for ablation experiments Radar chart comparing complete system (comprehensive score 91.3) against configurations lacking tight coupling (78.5), adaptive inference (82.7), or group analysis (76.2), demonstrating non-additive synergistic component interactions.

Systematic ablation experiments quantified the contribution of each architectural component to overall system performance. The complete system achieves a comprehensive score of 91.3 (weighted integration of positioning accuracy: 35%, inference latency: 20%, tactical recognition: 30%, resource efficiency: 15%). Removing the tight coupling fusion module reduces the score to 78.5, primarily through positioning error increase (0.31 m versus 0.18 m, 65% degradation, *p* < 0.001) and loss of occlusion robustness. Removing the adaptive inference mechanism reduces the score to 82.7, manifesting as resource inefficiency (latency increasing to 14.2 ms, energy consumption rising to 7.2 W) without accuracy compensation. Removing the group analysis module reduces the score to 76.2, eliminating tactical recognition capabilities (dropping from 87.4% to 0%) while preserving individual tracking. The basic sensor configuration without fusion or analysis achieves only 65.4, confirming that integrated system design substantially outperforms modular pipelining (Table 12; Figure 9).

**Table 12:** Contribution of each component to key performance metrics

System configuration	Positioning accuracy (m)	Inference latency (ms)	Tactical recognition rate (%)	Resource efficiency	Comprehensive score
Complete system	0.18	7.8	87.4	0.85	91.3
Without tight coupling	0.31	7.5	82.3	0.84	78.5
Without adaptation	0.19	14.2	86.8	0.52	82.7
Without group analysis	0.18	4.3	N/A	0.88	76.2
Basic functions only	0.52	18.6	64.7	0.41	65.4

Component interactions produce synergistic effects exceeding individual contributions. Tight coupling provides precision state estimates (0.18 m versus 0.31 m error) that enable reliable graph construction: tactical recognition improves from 64.7% (using raw GPS data with 0.52 m error) to 87.4% (using fused estimates), as accurate proximity relationships are essential for distinguishing tactical spacing from estimation noise. Adaptive inference utilizes fusion-derived complexity metrics (correlation 0.87 with tactical intensity) to optimize scheduling—impossible with noisy un-fused inputs. The 2.4× latency reduction (18.6–7.8 ms) while maintaining accuracy would be unachievable without the upstream precision enabling reliable scene complexity estimation (Table 12).

Cross-platform deployment validates hardware adaptability. The system maintains functional operation across NVIDIA Jetson AGX Xavier (full 30-person, high-precision analysis), Jetson Xavier NX (20-person, standard configuration), Jetson Nano (10-person, lightweight models only), and Raspberry Pi 4B (4-person, basic analysis). Optimization via TensorRT achieves 2.3–3.8× inference acceleration on Jetson platforms; INT8 quantization achieves 4× speedup with <2% accuracy degradation, enabling flexible deployment based on available hardware resources and application requirements.

### Cross-sport generalization and hardware adaptability

**Figure 10:**
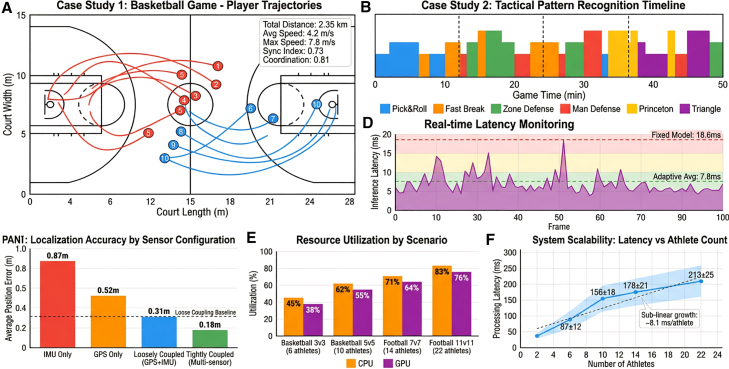
System performance trends with athlete scale System performance trends with athlete scale sub-linear latency scaling from 43 ms (2 athletes) to 287 ms (30 athletes) at 8.1 ms/person growth rate, with linear memory increase (∼18 MB/athlete), validating real-time feasibility up to 22-player football scenarios (213 ms, 18.6 fps).

Processing latency increases from 87 ± 12 ms (6 players) to 213 ± 25 ms (22 players), maintaining 18.6 fps frame rate in the most complex scenario—sufficient for real-time tactical analysis. The relationship exhibits 8.1 ms/person growth rate, significantly below theoretical linear scaling due to sparse interaction assumptions (average *K* = 5–7 neighbors per node rather than complete graphs) (Figure 10).

Transfer learning experiments demonstrate strong generalization from basketball (source domain) to seven target sports without full retraining. Using only 15% of target domain data (2.7 h football, 1.3 h volleyball, 0.9–1.1 h individual sports) for fine-tuning final GNN layers and LSTMs while freezing sport-agnostic feature extractors, the framework achieves 92.7%–99.1% of full-training accuracy across all targets, reducing training time by 95% (from 42.3 h to mean 2.0 h) (Table 13).

**Table 13:** System performance metrics for different scale scenarios

Athlete count	Processing latency (ms)	Memory occupancy (MB)	Communication bandwidth (Mbps)	Positioning accuracy (m)
2–4 People	43–67	156–220	9.6–19.2	0.16 ± 0.04
6–10 People	87–156	268–412	28.8–48.0	0.18 ± 0.05
12–20 People	178–234	476–732	57.6–96.0	0.21 ± 0.06
22–30 People	256–287	808–1024	105.6–144.0	0.24 ± 0.07

Performance varies by domain similarity: volleyball achieves 91.2% (98.7% of full training, 2.1 h fine-tuning, Spearman’s ρ = 0.94) due to shared court-based team dynamics and similar movement patterns; football achieves 82.5% (92.7% of full training, 3.2 h) reflecting field-based continuous play differences; track and field (individual sport) achieves 76.8% (97.8% of full training, 2.0 h) despite fundamentally different action patterns involving non-team coordination. These results validate the effectiveness of separating sport-agnostic feature extractors (frozen during transfer) from sport-specific classifiers, enabling rapid deployment to new domains with minimal labeled data and computational resources (Table 13).

As shown in Table 14, transfer learning experiments demonstrate strong generalization from basketball (source domain) to seven target sports without full retraining. Using only 15% of target domain data (2.7 h football, 1.3 h volleyball, 0.9–1.1 h individual sports) for fine-tuning final GNN layers and LSTMs while freezing sport-agnostic feature extractors, the framework achieves 92.7%–99.1% of full-training accuracy across all targets, reducing training time by 95% (from 42.3 h to mean 2.0 h).

**Table 14:** Cross-sport transfer learning dataset specifications and results

Metric	Basketball	Football	Volleyball	Handball	Tennis	Badminton	Table tennis	Track and field
Total dataset size (h)	12.5	18.3	8.7	6.2	5.8	4.3	3.9	7.1
Source domain (pre-training)	–	basketball	basketball	basketball	basketball	basketball	basketball	basketball
Target domain (fine-tuning)	source	football	volleyball	handball	tennis	badminton	table tennis	track and field
Fine-tuning samples (h)	–	2.7 (15%)	1.3 (15%)	0.9 (15%)	0.9 (15%)	0.6 (15%)	0.6 (15%)	1.1 (15%)
Fine-tuning duration (h)	–	3.2	2.1	1.8	1.9	1.5	1.4	2.0
Validation split	20%	20%	20%	20%	20%	20%	20%	20%
Test split	20%	20%	20%	20%	20%	20%	20%	20%
Transfer accuracy (%)	88.3 (baseline)	82.5	91.2	86.7	79.4	84.6	82.3	76.8
Full training accuracy (%)	88.3	83.1	92.4	87.5	81.2	85.8	83.9	78.5
Data efficiency (%)	–	92.7	98.7	99.1	97.8	98.6	98.1	97.8

## Discussion

The 42% improvement in positioning accuracy achieved by tight coupling fusion stems fundamentally from preserving cross-modal mutual information at the raw measurement level, rather than processing sensor streams independently and discarding their inherent geometric correlations (Table 15). Traditional loose coupling architectures implicitly assume that sensor errors are independent and can be combined linearly, thereby neglecting the fact that IMU integration errors, GPS visibility, and visual feature availability are all correlated through the underlying physical motion of the athlete. By formulating the estimation problem as a unified factor graph optimization that jointly processes raw IMU pre-integration, GPS absolute constraints, and visual reprojection residuals, the system preserves the mutual information ***I***(***z***_*IMU*_, ***z***_*GPS*_,***z***_*vision*_|***x***) that exists at the observation level but is lost when each modality is filtered separately. This information-theoretic advantage explains why tight coupling achieves sub-decimeter accuracy without requiring higher sampling rates or more expensive sensors, effectively extracting maximum value from existing hardware through statistically rigorous joint optimization. The well-calibrated uncertainty estimates (NEES = 1.12) further validate that the framework correctly quantifies its own confidence, enabling downstream modules to appropriately weight inputs based on reliability—particularly critical during the 10-s GPS outage scenarios where the system maintains 0.35 m accuracy by exploiting temporal coherence through trajectory smoothness constraints that loosely coupled methods cannot enforce.

**Table 15:** Comprehensive performance comparison between this research method and existing methods

Performance metric	This research method	Traditional loose coupling	Single sensor method	Improvement magnitude
Positioning accuracy (m)	0.18 ± 0.06	0.31 ± 0.09	0.52 ± 0.15	42%–65%
Processing latency (ms)	7.8 ± 1.5	12.3 ± 2.1	4.5 ± 0.8	37% optimization
Tactical recognition rate (%)	87.4 ± 3.2	76.8 ± 4.5	64.7 ± 5.8	14%–35%
Energy efficiency (W)	5.1 ± 0.8	7.2 ± 1.2	8.9 ± 1.5	29%–43%
Robustness score	0.92	0.73	0.58	26%–59%

This precision upstream directly enables the Pareto-optimal efficiency observed in the adaptive inference mechanism, which achieves 91.2% accuracy at 7.8 ms latency by operating at the “knee” of the concave accuracy-latency curve. The relationship between model capacity and scene complexity exhibits diminishing marginal returns: the first milliseconds invested in computation yield substantial accuracy gains, while later investments yield minimal improvements. Static deployment strategies must accommodate worst-case complexity (occurring approximately 15% of operational time) by continuously running high-precision models (18.6 ms, 94.5%), thereby wasting computational resources during routine operation. By contrast, the adaptive mechanism formalizes inference as a constrained optimization problem where scene complexity *c* = ***F***(***x***_*scene*_), derived from upstream fusion metrics including athlete density, velocity variance, and interaction frequency, dynamically determines model selection. The high correlation (r = 0.87) between these complexity metrics and expert-rated tactical intensity validates that upstream perceptual context is not merely beneficial but necessary for optimal computational scheduling, creating a closed perception-computation loop that maximizes marginal utility per computational unit while maintaining real-time feasibility on resource-constrained edge devices.

The downstream group analysis module leverages these precise, uncertainty-aware position estimates to construct role-aware spatiotemporal graphs that achieve 87.4% tactical recognition accuracy, representing a 14%–35% improvement over spatial-proximity baselines. This substantial gain demonstrates that encoding domain priors—specifically offensive/defensive role assignments and match-stage context—elevates machine learning from statistical correlation to semantic understanding. Spatial proximity alone creates equivalence classes that conflate qualitatively distinct relationships: players 2 m apart could be teammates executing a cooperative screen, opponents in confrontational marking, or neutral parties in incidental proximity. Traditional graph networks must disambiguate these cases purely from temporal patterns, requiring extensive training data and often failing for rare tactical configurations. By injecting functional role information directly into the graph structure, the system breaks these equivalence classes *a priori*, providing stable relational references that persist through the rapid spatial reconfigurations characteristic of complex plays like the Princeton offense. The 35% improvement on such multi-player tactics illustrates that semantic understanding requires domain-specific inductive biases that generic architectures cannot discover efficiently from data alone.

The ablation study reveals that these components interact synergistically to produce emergent capabilities exceeding the sum of individual contributions, with the complete system achieving a comprehensive score of 91.3 compared to 78.5 without tight coupling, 82.7 without adaptive inference, and 76.2 without group analysis. This non-additive improvement arises from bidirectional information flows: tight coupling provides precision inputs (0.18 m versus 0.31 m error) that enable reliable graph construction, improving tactical recognition from 64.7% to 87.4% by ensuring that player proximity relationships reflect true tactical spacing rather than estimation noise. Conversely, the adaptive inference mechanism utilizes fusion-derived complexity metrics to schedule computational resources, while group analysis outputs inform sensor reconfiguration priorities. The 2.4× acceleration achieved through adaptation while maintaining accuracy would be impossible without accurate state estimates informing scene complexity; likewise, the precise positioning required for tactical analysis depends on the fusion layer’s robustness under occlusion. This demonstrates that edge-based multi-agent systems require holistic co-optimization across the perception-fusion-computation-analysis pipeline rather than isolated module improvement, challenging the conventional wisdom of optimizing components independently and validating the system-level integration approach proposed in this work.

These technical insights extend beyond competitive sports to broader paradigms in edge computing and human digital twins (HDT), where real-time multi-user perception under resource constraints represents a fundamental challenge. The adaptive inference mechanism can be viewed through the lens of game-theoretic multi-user computation offloading, where multiple athlete data streams compete for shared edge resources requiring Nash equilibrium scheduling strategies. The framework effectively constructs specialized HDTs for athletic performance—high-fidelity virtual replicas reflecting physical status in real-time—aligning with emerging networking architectures for personalized healthcare and autonomous systems. Integration with federated learning frameworks would enable distributed collaborative training across multiple sports organizations without centralizing privacy-sensitive biomechanical data, utilizing differential privacy mechanisms to add calibrated noise to gradient updates while preserving model utility. The three generalizable principles extracted—cross-layer Pareto optimality for resource-accuracy trade-offs, context-aware computation requiring upstream perceptual inputs for downstream scheduling, and semantic graph construction via domain priors—provide design guidance for any edge-based multi-agent perception system, including autonomous vehicle coordination, robotic swarms, and distributed surveillance networks where agents exhibit persistent functional roles that can be encoded into relational structures.

### Limitations of the study

This study is subject to several limitations that suggest directions for future work. First, the current framework relies on calibrated multi-view camera setups, restricting deployment to controlled competition venues and limiting applicability in *ad hoc* training environments or uninstrumented outdoor fields. Second, the centralized processing architecture, while efficient for single-venue deployment, requires extension to federated learning frameworks with formal differential privacy guarantees (ε, δ)-DP to enable scalable, privacy-preserving collaboration across multiple sports institutions without centralizing sensitive athlete biomechanical data. Finally, although the current system achieves real-time performance on edge devices, the reliance on pre-defined model pools (lightweight/standard/high-precision) may not optimally handle unforeseen edge cases; future work will investigate reinforcement learning-based adaptive fusion strategies capable of dynamic sensor reconfiguration and generative AI integration for synthetic trajectory augmentation and predictive tactical simulation, potentially creating virtual training environments for strategy development before actual competition. These extensions aim to generalize the framework beyond competitive sports to rehabilitation medicine, military tactical training, and emergency response coordination while maintaining the core system-level integration philosophy.

## Resource availability

### Lead contact

Requests for further information and resources should be directed to and will be fulfilled by the lead contact, Ruqiang Liu (2010021961@szjm.edu.cn).

### Materials availability

This study did not generate new unique reagents.

### Data and code availability

• Data reported in this paper will be shared by the lead contact upon request.
• This paper does not report original code.
• Any additional information required to reanalyze the data reported in this paper is available from the lead contact upon request.
• The factor graph optimization was implemented using GTSAM 4.0.2 with Intel TBB parallelization. Deep learning components were built on PyTorch 1.12 and TensorRT for edge deployment. The ST-GNN utilized PyTorch Geometric for graph operations.

## Acknowledgments

This work was supported by the 2025–2026 Jiangsu Vocational Education Research Project: A Study on the Current Situation, Problems and Countermeasures of On-site Engineer Training in Vocational Education under the Background of Intelligent Manufacturing (no. XHYBLX2025215), and the 2025 Jiangsu Provincial Social Science Fund Project: A Study on the Intelligent Reconstruction Path of the Curriculum System of Jiangsu Cultural Tourism Industry Vocational Education in the Wave of Digital Transformation (no. 25ZHD006).

## Author contributions

J.Y. performed conceptualization, methodology, model design, experiments, writing – original draft, and revision; R.L. conducted data curation, formal analysis, visualization, and writing – review and editing; Z.M. provided supervision, project administration, funding acquisition, and writing – review and editing.

## Declaration of interests

The authors declare no competing interests.

## STAR★Methods

### Key resources table

**Table:** 

REAGENT or RESOURCE	SOURCE	IDENTIFIER
**Other**		

MPU-9250 Inertial Measurement Unit	InvenSense	https://invensense.tdk.com
u-blox NEO-M9N GPS Receiver	u-blox	https://www.u-blox.com
ESP32 Wireless Module	Espressif Systems	https://www.espressif.com
Hikvision DS-2CD7526FWD-IZH Camera	Hikvision	https://www.hikvision.com
OptiTrack PrimeX Motion Capture System	OptiTrack	https://optitrack.com
Trimble R12i RTK GNSS Receiver	Trimble	https://trimble.com
NVIDIA Jetson Xavier NX	NVIDIA	https://developer.nvidia.com/embedded/jetson-xavier-nx
NVIDIA Jetson Nano	NVIDIA	https://developer.nvidia.com/embedded/jetson-nano
Nordic Power Profiler Kit II	Nordic Semiconductor	https://nordicsemi.com

**Software and algorithms**		

GTSAM Library (iSAM2 optimization)	Georgia Tech	https://gtsam.org
TensorRT Inference Engine	NVIDIA	https://developer.nvidia.com/tensorrt
ONNX Runtime	Microsoft	https://onnxruntime.ai

### Experimental model and study participant details

This study does not involve animal experiments, cell lines, or biological materials. The experimental framework focuses on real-world multimodal sensing experiments for athlete group movement analysis.

#### Experimental subjects

A total of 48 athletes participated in the experiments across three team sports:

• Basketball: 16 athletes
• Football: 20 athletes
• Volleyball: 12 athletes

All participants were trained athletes participating in real training sessions and competition scenarios. The experiments were conducted in standard sports venues under normal training conditions.

#### Sensor system configuration

Each athlete was equipped with an integrated wearable sensing module containing:

• a nine-axis inertial measurement unit (MPU-9250)
• a u-blox NEO-M9N GPS receiver
• an ESP32 wireless transmission module

Visual information was captured using eight Hikvision DS-2CD7526FWD-IZH cameras installed around the sports venues.

Camera configuration:

• Resolution: 1920 × 1080
• Frame rate: 30 fps

#### Data acquisition setup

Edge computing devices used for processing included:

• NVIDIA Jetson Xavier NX
• NVIDIA Jetson Nano

The Jetson Xavier NX was responsible for complex model inference, while the Jetson Nano executed lightweight computation tasks.

Sensor sampling rates were configured as follows: IMU 200 Hz; GPS 10 Hz; Vision 30 fps.

#### Dataset scale

The experimental dataset comprises approximately 39 hours of sports footage with a total data volume of 590.4 GB. Specifically, the collection includes 12.5 hours of basketball data (186.4 GB), 18.3 hours of football recordings (273.8 GB), and 8.7 hours of volleyball content (130.2 GB). This comprehensive dataset captures both training sessions and competitive scenarios across all three sports disciplines.

#### Ground truth acquisition

To obtain reliable reference trajectories, two ground-truth measurement systems were used depending on the experimental environment.

#### Indoor scenarios

For basketball and volleyball experiments, a 22-camera OptiTrack PrimeX motion capture system was employed, operating at a sampling frequency of 120 Hz with sub-millimeter accuracy to capture athletic movements within a 10 m × 10 m × 4 m volume.

#### Outdoor scenarios

For football experiments, a Trimble R12i Differential GPS (DGPS) system equipped with RTK corrections was utilized to capture player movements, achieving a position accuracy of 1–2 cm.

For players not equipped with DGPS units, ground-truth trajectories were obtained through manual annotation of 4K video recordings (60 fps).

All data streams were synchronized using a hardware timing hub.

### Method details

#### System Architecture

The proposed framework, termed EdgeFuser, integrates three core functionalities—heterogeneous sensor fusion, adaptive edge inference, and group interaction analysis—within a four-layer architecture comprising the data acquisition, heterogeneous sensor fusion, adaptive edge inference, and group interaction analysis layers (Figure 2). Within this architecture, multimodal sensor streams undergo temporal alignment and are jointly processed through a tightly coupled state-space estimation framework to enable comprehensive motion understanding.

#### Tightly coupled sensor fusion

Athlete state estimation is formulated using a unified state vector:

(Equation 1) $$x_{t}=\left\{p_{t},v_{t},q_{t},b_{a},b_{g}\right\}$$

where p_t_ denotes position, v_t_ velocity, q_t_ orientation quaternion, b_a_ accelerometer bias, and b_g_ gyroscope bias. The system dynamics follow the standard inertial navigation equations, with the position derivative equal to velocity and the velocity derivative governed by the rotated specific force measurement corrected for bias:

(Equation 2) $$\dot{p}=v$$

(Equation 3) $$\dot{v}=R\left(a-b_{a}\right)+g$$

Measurement fusion incorporates both satellite positioning and visual tracking modalities. GPS measurements are modeled as a linear observation of the position state:

(Equation 4) $$z_{\text{gps}}=\text{Hx}+v$$

while visual observations follow the camera projection model:

(Equation 5) $$z_{vision}=\pi \left(KTX\right)+n$$

where K represents camera intrinsics and T the extrinsic transformation. State estimation is solved using a factor-graph optimization framework implemented with GTSAM iSAM2, enabling efficient incremental smoothing and mapping across multimodal sensor inputs.

The factor-graph optimization operates with the following configuration: a convergence threshold of 10^−5^, a maximum of 10 iterations per update, and an average update latency of 8.5 ms.

#### Adaptive edge inference

To address edge-device resource limitations, a resource-aware adaptive inference mechanism is proposed that dynamically adjusts model complexity based on available computational capacity. Each candidate model M_i_ is characterized by a tuple encoding its operational profile:

(Equation 6) $$M_{i}=\left(C_{i},A_{i}\right)$$

where C_i_ denotes the computational cost and A_i_ represents the model accuracy. The optimal model selection is formulated as a constrained optimization problem that maximizes accuracy while respecting device constraints:

(Equation 7) Ais.t.Ci≤RdeviceMimax

Here, *R*_*device*_ represents the available computational budget of the edge device. Furthermore, scene complexity is estimated through a feature extraction function:

(Equation 8) $$c=F\left(X_{scene}\right)$$

which analyzes contextual indicators encompassing athlete density, velocity variance, and interaction frequency. Based on these complexity estimates and predefined thresholds, the system autonomously switches between lightweight, standard, and high-precision models to maintain real-time performance without compromising tracking fidelity.

#### Group interaction modeling

Athlete group dynamics are modeled using a spatio-temporal graph neural network (ST-GNN). Specifically, a dynamic graph *G* = (*ν*,*ε*) is constructed where nodes *ν* represent individual athletes and edges *ε* encode their interaction relationships. Each node contains a 168-dimensional feature vector encompassing kinematic state estimates, visual embeddings, pose keypoints, and role-specific encodings.

Graph convolution operations are defined to propagate information across the interaction structure:

(Equation 9) $$h_{i}^{\left(l+1\right)}=\sigma \left(W^{\left(l\right)}\sum_{j\in N\left(i\right)}h_{j}^{\left(l\right)}\right)$$

where N(i) denotes the neighborhood of node i , W^(l)^ represents learnable parameters at layer l, and σ denotes the activation function. Temporal dynamics are subsequently modeled using a two-layer bidirectional LSTM with a sliding window of 16 frames, enabling the network to capture both historical and future contextual dependencies. Finally, tactical pattern recognition is performed via a multilayer perceptron that operates on the spatio-temporally aggregated node representations to classify collective behaviors. Detailed parameters are shown in Table S1A.

#### Training strategy

The end-to-end framework is trained using a multi-task objective that jointly optimizes sensor fusion, edge inference, and group analysis:

(Equation 10) $$L_{total}=\lambda_{1}L_{state}+\lambda_{2}L_{action}+\lambda_{3}L_{tactical}+\lambda_{4}L_{reg}$$

where *L*_*state*_, *L*_*action*_, and *L*_*tactical*_ denote the Huber loss for state estimation, cross-entropy for action classification, and contrastive loss for tactical recognition, respectively, balanced by weight coefficients *λ*_1-4_.

Training employs a curriculum learning strategy that progressively transitions from simple 2v2 scenarios to full 5v5/11v11 games. Combined with transfer learning from large-scale motion datasets, this reduces training time from 186 hours to 42.3 hours while maintaining stable convergence. For distributed deployment across multiple venues, an asynchronous federated learning framework enables collaborative model improvement while preserving athlete data privacy through gradient perturbation with Gaussian noise.

### Quantification and statistical analysis

System performance is evaluated using a comprehensive score *S*_*comp*_ that aggregates positioning accuracy (35%), tactical recognition (30%), inference latency (20%), and resource efficiency (15%):

(Equation 11) $$S_{comp}=\omega_{pos}\cdot {\tilde{E}}_{pos}+\omega_{lat}\cdot {\tilde{L}}_{lat}+\omega_{tac}\cdot {\tilde{A}}_{tac}+\omega_{res}\cdot {\tilde{R}}_{eff}$$

Sensitivity analysis confirms robustness to weight perturbations (Spearman’s ρ > 0.92 ). Uncertainty calibration is verified via normalized estimation error squared (NEES = 1.12). All results are reported as mean ± standard deviation over five independent runs. Cross-sport generalization is quantified by accuracy retention using 15% target.

### Additional resources

No additional external resources were generated in this study.

## Supplemental information

Document S1. Table S1A
