# Supplementary material for: EdgeFuser: A tightly coupled adaptive framework for real-time athlete group analytics
Source: iScience. 2026 Apr 1;29(5):115577. doi: 10.1016/j.isci.2026.115577 (PMC13138219; doi:10.1016/j.isci.2026.115577)
Supplement: Document S1. Table S1A [file mmc1.pdf]

**iScience, Volume *29***

## Supplemental information

# EdgeFuser: A tightly coupled adaptive framework for real-time athlete group analytics

**Juan Yang, Ruqiang Liu, and Zhenyu Miao**

**Table S1. Comprehensive ST-GNN Architecture Hyperparameters**

| Component | Parameter | Value |
|---|---|---|
| **Input Features** | Node feature dimension | 168 |
| | Feature composition | Kinematic (6) + Appearance (128) + Keypoints (34) + Role (4) |
| | Temporal window size T*T* | 16 frames (0.8s) |
| | Update frequency | 20 Hz |
| **Spatial GNN** | Number of graph conv layers | 3 |
| | Hidden dimensions | [168→256, 256→256, 256→128] |
| | Attention heads per layer | [4, 4, 2] |
| | Attention type | GATv2 |
| | Aggregation method | Concatenate (layers 1-2), Average (layer 3) |
| | Activation function | LeakyReLU (negative slope 0.2) |
| | Edge construction threshold dthreshold*d*threshold | 5m (basketball/volleyball), 8m (football) |
| | Max neighbors per node K*K* | 8 |
| **Temporal LSTM** | Number of LSTM layers | 2 |
| | LSTM type | Bidirectional |
| | Hidden dimensions | Layer1: 128 per direction, Layer2: 64 per direction |
| | Dropout rates | Layer1: 0.3, Layer2: 0.2 |
| | Output temporal dimension | 256 (concatenated final states) |
| **Classification Head** | MLP layers | [256→128, 128→64, 64→C] |
| | MLP activations | ReLU |

| Component | Parameter | Value |
|---|---|---|
| | MLP dropout | 0.3 (first layer only) |
| | Output classes C$C$ | 12 (basketball), 15 (football), 8 (volleyball) |
| **Training** | Optimizer | AdamW |
| | Learning rate | 1e-3 (initial), cosine decay to 1e-5 |
| | Batch size | 32 |
| | Gradient clipping | 1.0 |
| | Weight decay | 1e-4 |
| | Total trainable parameters | 2.8M (lightweight) to 8.2M (full) |
| **Regularization** | Dropout (spatial) | 0.2 (applied after each GNN layer) |
| | Dropout (temporal) | As specified above |
| | Batch normalization | Applied after each GNN layer |
| | Label smoothing | 0.1 for classification loss |
| | Early stopping patience | 15 epochs |
| | Model selection | Best validation accuracy |
